# “It may also have prevented churchgoers from falling asleep”: southernwood, *Artemisia abrotanum* L. (fam. Asteraceae), in the church bouquet, and its contemporary presence as a heritage plant in Sweden

**DOI:** 10.1186/s13002-020-00401-4

**Published:** 2020-08-28

**Authors:** Ingvar Svanberg, Erik de Vahl

**Affiliations:** Uppsala Universitet, Uppsala, Sweden

**Keywords:** Church plants, Cultivated plants, Heritage plants, Historical ethnobotany, Ritual plants, Seasoning

## Abstract

**Introduction:**

Southernwood, *Artemisia abrotanum* L., is a plant that has been cultivated for centuries. Most probable is that it has its origin in the eastern Mediterranean area. It has been kept for its fragrance and has a history of being a medicinal and insect-repellent plant. In earlier centuries, the plant was commonly found in peasants’ gardens in Sweden and utilised especially as a component in the bouquets brought to church by women. The aim of this article is to bring together data about *Artemisia abrotanum* and to summarise its cultural history and folk botanical importance. In Sweden, it is still grown in some gardens in the countryside and is esteemed for its fragrance.

**Methods:**

In the early twentieth century, various folklore archives in Sweden (Lund, Uppsala) distributed questionnaires about the use of church bouquets. These records provided interesting information about the importance of southernwood and other species. We have also used data found in ethnographic records and local historical reports. Between 2007 and 2017, a nationwide inventory organised by the Programme for Diversity of Cultivated Plants (POM) documented and gathered several heirloom varieties of southernwood.

**Results and discussion:**

Together with a few other domestic plants of foreign origin (e.g. *Lavandula angustifolia* Mill., *Tanacetum balsamita* L., and *Tanacetum vulgare* L.), *Artemisia abrotanum* has been cultivated throughout Sweden in peasants’ gardens as a medicinal plant and for its fragrance. According to the sources, *Artemisia abrotanum* was one of the most common species cultivated by the Swedish peasantry. Although used in folk medicine and to some extent as a repellent, it was most esteemed for its fragrance. Peasant women would carry a twig of the plant in the obligatory church bouquet or in the hymnal when attending the services in the Lutheran church on Sundays. In Sweden, this custom with the church bouquet has been known since the time of the Reformation in the sixteenth century and survived until the late nineteenth century, when major changes took place in connection with industrialisation, modernisation, secularisation and urbanisation. Although the custom with the church bouquet disappeared, nationwide inventories conducted by the Programme for Diversity of Cultivated Plants in 2007–2015 revealed that the plant still exists in many gardens on the countryside throughout Sweden as a cultural relict and reminiscence plants. People care for the plant, have sentiments for it and it is spread from person-to-person. Several heirloom varieties have been discovered, which will be preserved ex situ for the future.

**Conclusions:**

Southernwood was probably the most commonly used herb in the peasant women’s church bouquet until the end of the nineteenth century. It had a satisfying fragrance and was easy to grow. Although the custom has disappeared, the plant has survived until the present day in many gardens as a reminiscence of the custom of former times.

## Introduction

The pre-industrial peasant society in Sweden (i.e. before the 1880s) is frequently described as consisting of hardworking, modest people without splendour or extravagance. However, as pointed out by the Swedish author Carl Fredrik Dahlgren in 1831, many peasant women had a shared interest in fashion. As examples, he mentions younger peasant women who attended church in silk or saffian shoes with thin cotton stockings, even in wintertime. They carried hand fans and luxurious “church spices” (i.e. cloves, ginger, sugar). Others carried scented plants wrapped in a handkerchief or in the hymnal [1, 2].

In earlier times, bouquets of fragrant plants and beautiful flowers of the season were important additions to feminine fashion during Sunday services in the Swedish Lutheran church. Until the beginning of the twentieth century, it was a widespread custom among women to carry floral bouquets in their hands or to insert flowers into the hymnary when attending church services [2]. In addition to their use as food or in remedies, the usage of plants in folk customs and religious rituals is one of many bio-cultural domains of interest for ethnobiologists. Cultivated plants in particular were used in church rituals by the Swedish peasantry. This demonstrates that flowers and herbs could have attractive, emotional and spiritual functions in the peasant society [2].

According to British anthropologist Jack Goody, plants have always had a diverse use and symbolic significance in Christian church tradition [3]. Ethnobotanist Łukasz Łuczaj gives an interesting example from southeastern Poland where priests blessed bouquets brought to Catholic **churches** on the Assumption Day [4, 5]. There are of course a multitude of examples of plant use and floral decorations within church rituals from elsewhere within the Christian world [3, 6].

**Fig. 1:**
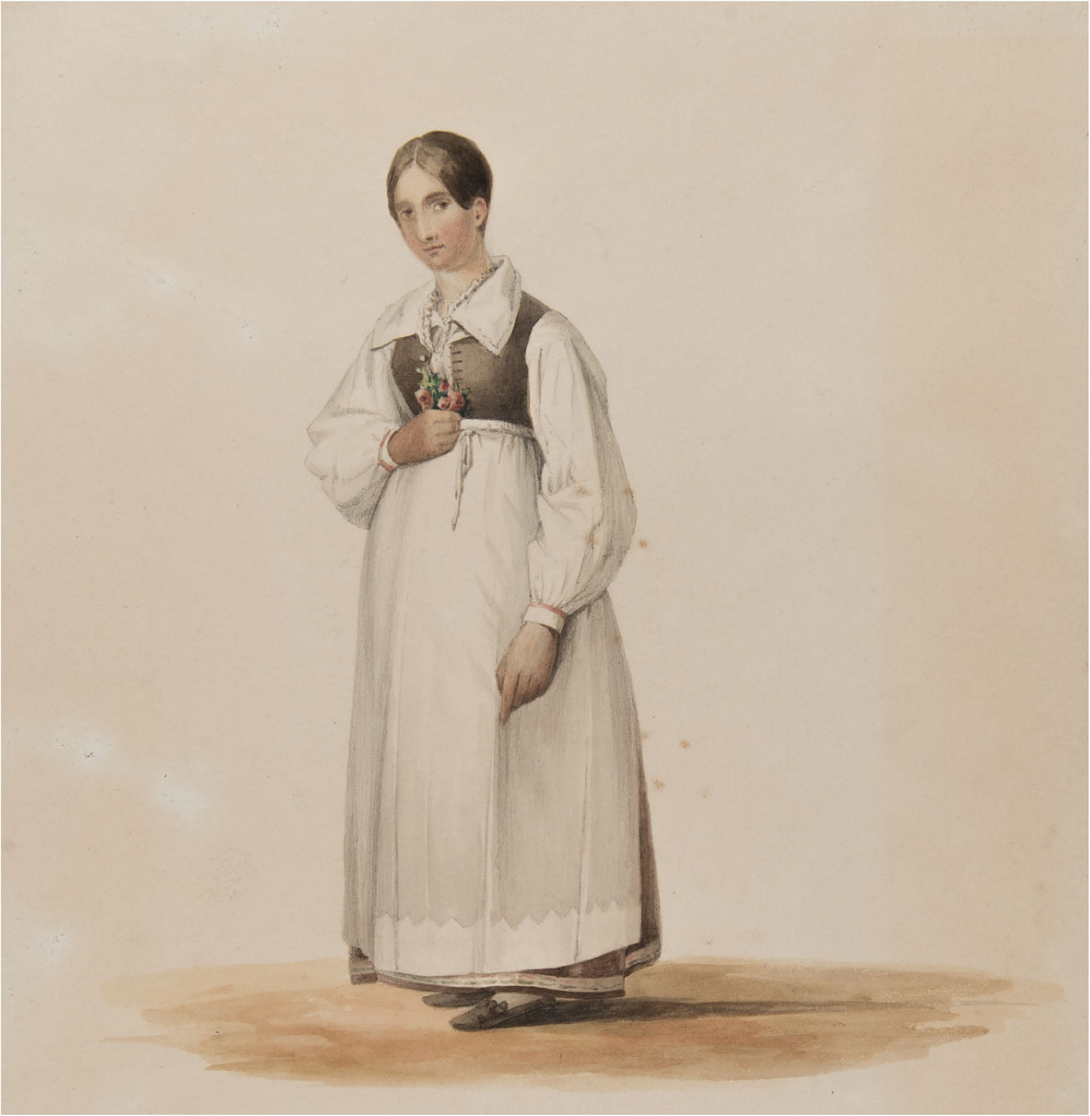
Peasant woman in Blekinge with a church bouquet (Watercolour by Joseph Magnus Stärck 1840; Courtesy The Nordic Museum, Stockholm)

We can still find plants and floral decorations in Swedish Lutheran churches. Plants are used as altar flowers, including pussy willow branches, *Salix caprea* L., on Palm Sunday; bouquets and flowers are used in connection with church funerals, and bouquets as part of church rites (e.g. bridal bouquets), and as decoration on the graves in the churchyard [2, 6]. Floral presence in the church context, including the earlier Swedish tradition of scented herbal bouquets carried by women during services, was part of the devout or pious life of the pre-industrial society [2, 7] (Fig. 1).

The custom of church bouquets has been recorded in Sweden since the late sixteenth century, i.e. from the time of the Reformation in the mid-sixteenth century in Sweden [8]. It was, for instance, mentioned in a book published by the royal secretary, Laurentius Johannis Lælius, in 1591 [9]. In 1665, proverb compiler Christopher Grubb recorded the phrase “Not all flowers can be used in a church bouquet”, which testifies that this was a well-established custom at the time [10].

Several scented plants were used in the church bouquet, for instance *Lavandula angustifolia* Mill.., *Tanacetum balsamita* L., and *Tanacetum vulgare* L. (see Table 1). However, the most common, according to folkloristic records, was the southernwood, *Artemisia abrotanum* L. (Fam. Asteraceae). It was a plant cultivated in almost all parts of Sweden, the exception being the province of Lapland.

**Table 1:** Species in the church bouquet at the end of the nineteenth century

Species	Number of records
**Cultivated plants**	
*Artemisia abrotanum* L.	45
*Lavandula angustifolia* Mill.	30
*Tanacetum balsamita* L.	28
*Tanacetum vulgare* L.	12
*Thymus vulgaris* L.	9
*Hyssopus officinalis* L.	8
*Melissa officinalis* L.	5
*Armoracia rusticana* P.G.Gaertn., B.Mey. & Scherb.	1
**Others**	
*Anthoxanthum odoratum* L.	3
*Galium verum* L.	1
*Haploporus odorus* (Sommerf.) Bondartsev & Singer	1

Source: Questionnaire ULMA Frågekort 30 “Kyrkbuketter” distributed in 1930

Southernwood, *Artemisia abrotanum* L. (fam. Asteraceae), is an old-fashioned herb, which has probably been grown as a medicinal and aromatic plant in Europe since the Roman Iron Age [11, 12]. It is explicitly referred to as present in Sweden in medieval and early modern written sources [13–16]. However, although it can survive in locations where it has been cultivated for a long time (even centuries), it has almost never become naturalised in Sweden [17, 18]. It is still grown as an herb in some Swedish rural gardens, although very seldom used for folk medicinal purposes or as seasoning. Instead, it is nowadays grown as a cultural relict—a kind of dear memory—from earlier days when Swedes were regular churchgoers and not secular as they are today. People still care for the plant and have sentiments for it, and it is spread from person-to-person [19, 20].

## Aim of the study

The purpose of this study is to discuss the cultural history of the use of *Artemisia abrotanum* as a garden plant in Sweden since the medieval times. We will especially illuminate its use in the traditional bouquet that churchgoing women would bring with them when attending services. It is a study of the interest of Swedish peasant women in folk botany. Furthermore, we will discuss the pre-industrial custom of including southernwood and other floral species in the church bouquets and its gradual regression in connection with the social changes that took place in connection with industrialisation and modernisation. We will also examine how the documentation and findings of *A. abrotanum* from the Swedish national inventories for cultivated plants correspond to older ethnobotanical records and literature. Nowadays, when most secular Swedes hardly ever attend church, why is southernwood as a cultural relict plant still cared for in some rural gardens? Finally, we will discuss the values of the preserved plant material as part of a cultural heritage.

## Methods and sources

Our knowledge concerning old cultivated plant species in Sweden is still very limited [2, 18]. However, a thorough review of various written and unpublished sources, a consistent contours comparative perspective and consideration of careful source criticism can help to distinguish some and even certain patterns. An intense search for references relating to the traditional use of *A. abrotanum* in pre-industrial society was performed in herbals, floras and ethnographic records from Sweden [21]. We have also used the questionnaires that were distributed in the first part of the twentieth century by Lund University’s Archives of Ecclesiastical History (LUKA 1 “Kyrklig folklivsforskning” 1942), the Folklife Archives at Lund University 1941 (LUF Sp. 111 “Kryddor”) and 1952 (LUF 88 “Kyrkkryddor”), and the Institute for Language and Folklore, Uppsala (former Uppsala Dialect and Folklore Archives), Uppsala, in 1932 (ULMA Frågekort 30 “Kyrkbuketter”) [22].

According to a nationwide survey that the senior author (IS) conducted in 2019, which is planned to be discussed further in a forthcoming paper, no contemporary cultural uses of *Artemisia abrotanum* was recorded whatsoever. However, the present study uses documentation collected in a nationwide inventory that was organised by the Programme for Diversity of Cultivated Plants (POM). Several heirloom varieties of southernwood were documented, collected and evaluated in cultivation trials between 2007 and 2017. The plant material was documented together with records of cultivation history, use and traditions. The most interesting materials with documented history from before 1950 were selected for preservation in the Swedish National Gene Bank at the Swedish Agricultural University in Alnarp [23]. These accessions have been morphologically described, and one accession has also been introduced into cultivation as a cultivar [20]. In the POM inventories 2007–2017, data on contemporary uses of *A. abrotanum* in Sweden are scarce, even though several remaining plant specimens in old gardens have been documented. The owners of these gardens had vivid recollections of the previous generations’ stories about the plant [20].

The method we have used is called source pluralism. It is described by the historian Janken Myrdal as the use of diverse scattered historical source material as a way to describe phenomena that have left few and scattered traces in records [24]. By combining records from written source responses from questionnaires as well as documentation from inventories and cultivations trials, we have tried to deepen our knowledge of an obscure garden plant mainly grown by the peasantry. Garden historians have earlier used similar methods. For instance one study describing the earliest gardens of Övedskloster estate in Skåne [25] as well as recent research into the supply of commercial plants in market gardens in Sweden from 1900 to 1950 [26]. When researching species traditionally grown or used by the peasantry in pre-industrial settings, the use of questionnaires as a source is particularly valuable [21].

### Southernwood

**Fig. 2:**
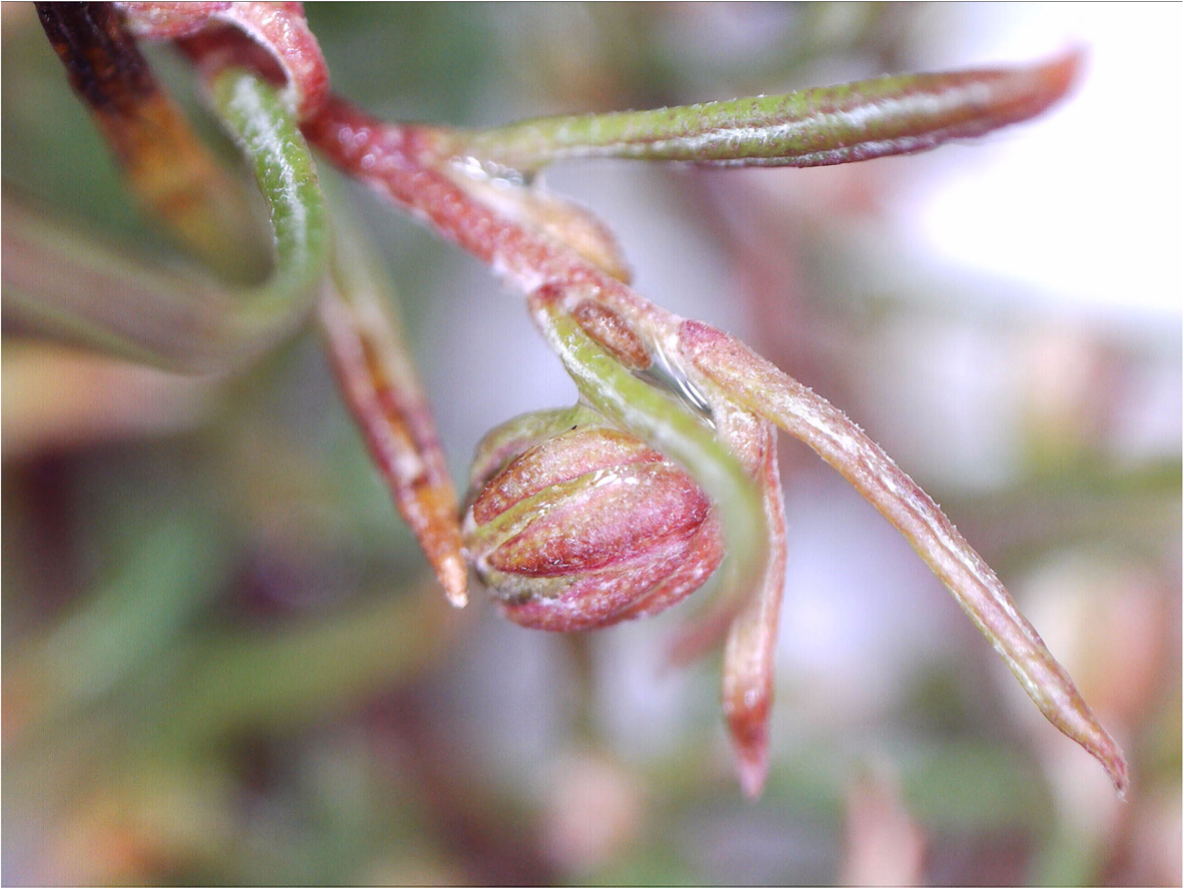
Flower-bud of *A. abrotanum* “Predikoväcka” in mid-October 2019 pre-inflorecence in southern Sweden (Photo Erik de Vahl)

**Fig. 3:**
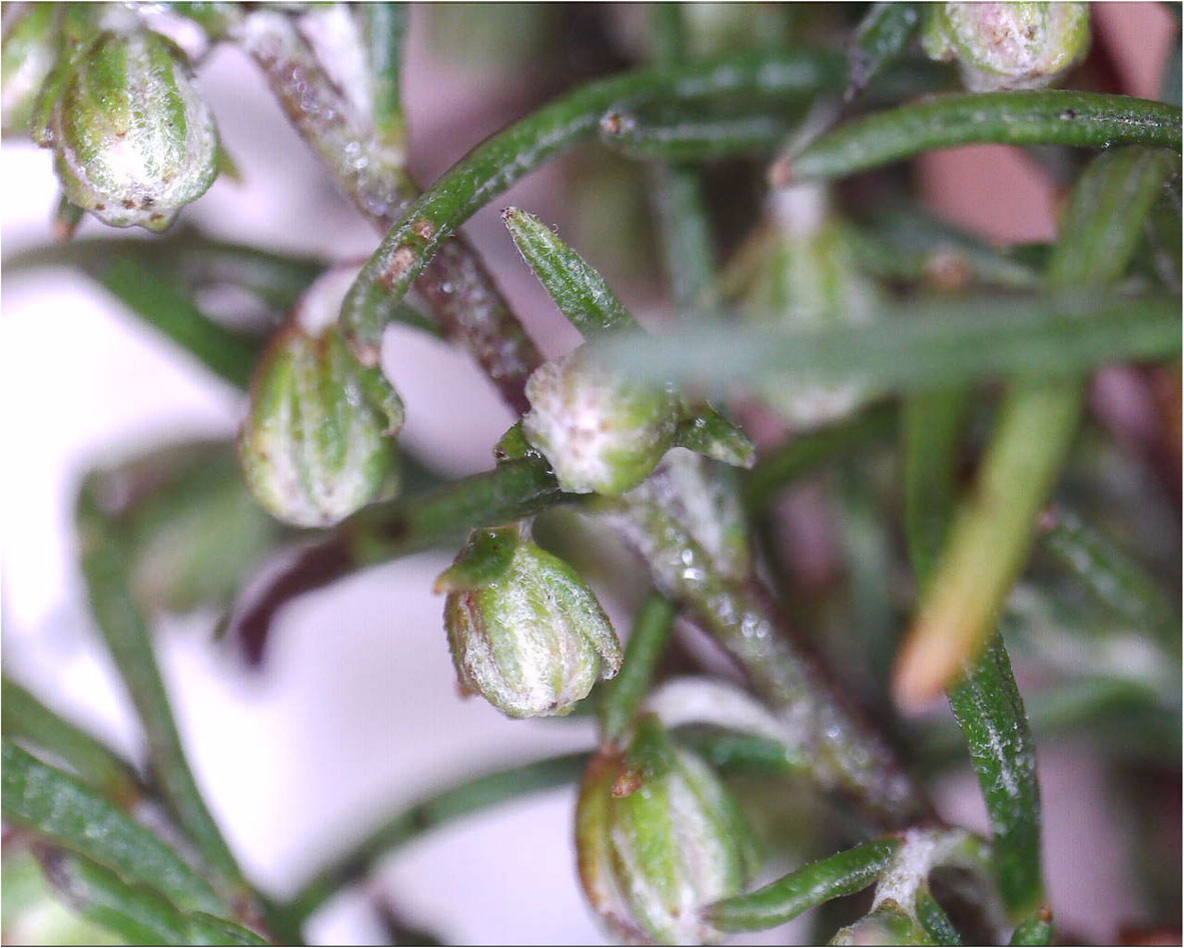
Flower-bud of *A. abrotanum* “Söbben” in mid-October 2019 pre-inflorecence in southern Sweden (Photo Erik de Vahl)

*Artemisia abrotanum* is a perennial plant with highly aromatic leaves [11, 20]. It forms a small bushy shrub and is widely cultivated in Europe. The slender leaves, which resemble those of dill, grow along the plant’s stiff stems. Southernwood can grow to be from 50 to 130 cm high, and from 30 to 60 cm wide. When touched, it gives off a scent with varying compositions of lemon, tangerine and camphor for different cultivars or genotypes. Its inflorescences, which are small and yellowish, appear during autumn. In northern Europe, there is never enough time for them to fully open (Figs. 2 and 3). However, the plant can easily be propagated with cuttings or by dividing the roots [11, 20].

The original habitat of *A. abrotanum* is obscure, but it is supposed to grow wild in the eastern Mediterranean and probably has its origins in western Asia [11, 12, 20]. Its contemporary common English name comes from the Old English *sûðerne wudu* ‘southernwood’, that is, a ‘woody plant that comes from the south’ [27]. Its native range nowadays covers Armenia, the Caucasus, Turkey, Ukraine, southern Russia and part of the Balkan Peninsula [11].

References to its use as a cultivated medicinal herb are found in the pre-modern literature. It is mentioned as *abrotanum* in the plant catalogue featured in *Capitulare de villis vel curtis imperii*, the edict issued by Charlemagne in 812 to regulate the administration of his crown estates. This catalogue indicates its range as a cultivated plant [11, 28]. Also, the Alemannic Benectine monk Walafrid Strabo (c. 808–849), who lived on Reichenau Island in southern Germany, mentions it as a repellent plant in his *Liber de cultura hortorum* from around 840 [29]. The herb seems to have spread over Europe during the Migration and Middle Ages, probably with the expansion of monastic gardens, and was used in monastic medicine. Its presence in northern Europe and the British Isles is recorded from the mid-sixteenth century. European immigrants brought it to North America in the early seventeenth century [11].

Southernwood contains up to 1.4% essential oil, chiefly absinthol. Minor components include fenchene, sabinene, alpha-caryophyllene, and beta-caryophyllene. The plant also contains abrotanin, a bitter alkaloid that is active against bacteria [11].

## Results

### Historical context

In the mid-nineteenth century, the population of Sweden was mostly rural with around 90% of the population still living in the countryside in 1850. Sweden’s industrialisation and urbanisation was rather late compared to many other West European nations. Cities only began to grow in the 1880s and 1890s, when industries were established, infrastructures were modernised and the demand for a labour force increased. Simultaneously, Sweden had a large trans-Atlantic emigration [30]. Rural areas were abandoned, and by 1930, about 50% of the population lived in cities. Today (2020), almost 88% of Swedes are urbanites [31].

With urbanisation came modernisation and cultural changes. The socio-cultural implications were extensive. Old customs of the pre-industrial peasant society began to fade away, as new consumption patterns increased, social control softened, the influence of the Lutheran church lost ground, and new political ideas were introduced. New attitudes, norms and practices replaced old ways [7]. The urban working class and eventually the middle class were increasingly secular, individualistic and modern. Their attitudes too and needs for plants differed from those of the pre-industrial peasantry in their new habitats [32].

### Historical use

Southernwood is mentioned in medieval medical books and herbals [11, 16, 33]. From Swedish medieval sources, Inger Larsson lists its use as a treatment for sleep talking, female diseases, and in animal medicine [33]. In Johannes Franckenius’ *Speculum Botanicum* from the early 1600s, *Artemisia abrotanum* was called *abrodd mann* “male southernwood” and shared its Swedish name with cotton lavender, *Santolina chamaecyparissus* L., also called *abrodd qwinna* “female southernwood” [34]. These early names are also known from Denmark [35, 36]. Most of its Swedish folk names from medieval times and onwards (*abrot*, *obroth*, *aabrut abrott*, *abrodd*, contemporary *åbrodd*) are derived from its pre-Linnaean Latin name *abrotanum* (originally from Ancient Greek ἀβρότονον) [16, 37].

The plant has been widely used within European folk botany and local medicine [37]. The early herbals mentioned its use as a medicinal plant, treatment for impotence, spice, herbal tea, and as insect repellent in continental Europe and among immigrant communities in North America. It was also used as an ethnoveterinary remedy to treat horses and sheep [11, 37–41]. On the other hand, concerning its usage there only exists a limited number of similar folk botanical data in Swedish ethnographic literature. For instance, the blacksmith Gustaf Ericsson describes how Swedish peasants in the province of Södermanland in the early 1800s would put southernwood in their wardrobes in order to repel moths [42]. In the province of Uppland, it was reported that southernwood was beneficial in healing wounds [43].

It has been mentioned in the publications of Schola Medica Salernitana since the twelfth century [44]. Its diverse utility in the preparation of medicaments is confirmed as early as the sixteenth century Swedish-Danish medicinal books [14, 14]. European pharmacopoeias have long recommended the use of southernwood for making remedies. In the Swedish Pharmacopoeia, it is listed in the various editions from 1775 to 1874 [44]. While it was particularly prized as a diaphoretic, it also was used to treat liver, spleen and stomach problems [11, 44]. In Johannes Palmberg’s herbal from 1684, southernwood is described as a cure for hair loss and amnesia [45]. The lectures of Johannes Franckenius, professor in medicine at Uppsala University from the 1640s, discussed the many medicinal virtues of southernwood [46]. There are also a few records of its use in folk medicine. According to a report, the inhabitants of the province of Skåne crushed the plant and applied it to boils [47]. Other treatments consisting of southernwood are also mentioned in the ethnographical literature [42].

### Its use in the church bouquet

The church bouquet was an important female attribute in the church. A grown-up woman could hardly arrive at the church service without a bouquet in her hand, or at least a scented twig in the hymnal. Southernwood was probably the most common species in this context (with the exception of the province of Gotland, where *Lavandula angustifolia* Mill. was the most common species). It was grown in the peasant garden, and its fragrance kept the churchgoer awake. It also had the advantage of being sustainable [42, 48, 49]. The amateur botanist, clergyman and local historian, Erik Modin, stated that southernwood was known as a church herb in the province of Härjedalen [50]. Another priest, Georg Bergfors, also reports from Ångermanland that the twigs of southernwood were brought to church during summer for their fresh and pleasant scent. He adds that its scent may also have prevented churchgoers from falling asleep [51].

Another account describes how young women from Blekinge would compete with each other on their way to church to find the best-smelling southernwood to tease and impress the men by tickling the men’s noses with bouquets. The respondent stresses that there could be quite a difference in scent between the plants but that this was solely a result of different growing locations. Even though this might have been the common belief, inventories showed that, historically, different genotypes certainly have been spread and grown in rural areas throughout Sweden. The ethnographic literature points out that southernwood were common in the countryside, even in the far north of Sweden, during the early 1900s [20, 50–52].

Documentation of the church bouquet and the practice of bringing different herbs to the services might give us an insight into older knowledge tied to plants and their uses. Besides southernwood, other common herbs known to be in use in the church bouquet were common lavender, *Lavandula angustifolia* Mill., costmary, *Tanacetum balsamita* L., garden thyme, *Thymus vulgaris* L., spearmint, *Mentha spicata* L., hyssop, *Hyssopus officinalis* L., common balm, *Melissa officinalis* L., and tansy *Tanacetum vulgare* L., [2, 7, 52–57]. The most common species used in the church bouquet in the province of Gotland were *Lavandula angustifolia* Mill., *Thymus vulgaris* L., *Matthiola incana* (L.) R. Br., *Tanacetum vulgare* var. *crispum* L. and *Hyssopus officinalis* L. in the province of Härjedalen, while in Norrbotten the women used scented grass [58].

These were all cultivated species grown widely by the peasantry and also had a wide range of uses outside the church. Every household could manage to grow at least some of these herbs in its small allotment, along the village street, and in the peasant garden [2, 53, 54, 56].

### Ornamental plants used in the church bouquet

However, what the church bouquet contained was not limited to scented plants. Flowers considered beautiful were also important in bouquets, especially those of younger women. The interest in and use of ornamental flowers was certainly an influence, first from the Baroque Period and later from the Romantic Period. The ethnographic reports and responses to questionnaires show that girls in springtime gladly included *Hyacinthus orientalis*, *Tulipa gesneriana* and *Narcissus pseudonarcissus* in their bouquets. These are all well-known cultivated plants from the peasant allotments and gardens [2]. When the bird cherries, *Prunus padus*, were flowering, women put some flowers in their hymnbooks [59]. On Whitsunday 1834, one observer in Uppsala noticed that girls coming from outside of town brought countless *Fritillaria meleagris*, L. These grew in large quantities on a meadow south of the town, which the girls passed on their way to church. After the service, he saw the flowers spread on the streets [60]. In the early summer of 1749, Linnaeus noted stalks of *Fritillaria imperialis* L. in a church in the eastern part of the province of Skåne [61]

Later, they could bring flowers of *Syringa vulgaris* L. and *Convallaria majalis* L. The red peonies, *Paeonia officinalis* L., were in bloom for at a time when the churches celebrated special youth holidays [59, 61]. They were thus assigned a special virtue in the play between the sexes [2]. When botanist Samuel Liljeblad visited Karlstad in 1797, he noticed that young peasant women at rural churches carried bouquets of *Epilobium angustifolium* L. [62].

As the summers progressed, the species in the bouquets were changed to new ones. Around midsummer, women carried branches of lavender and carnations in their hand when they attended church, according to one record from Loshult parish in Skåne. Of course, the splendour of flowers increased in summer and declined in autumn [63]. Houseplants also came into use [2].

In this way, the composition of bouquets varied during the year, giving new experiences, new floral scents and new colours. After the service, flowers could be placed on a relative's grave or taken home to keep in a box for the scent. Plants that were prone to wither quickly were left in the benches, much to the annoyance of the churchwarden [60, 63, 64].

### Instead of bouquets

As observed, southernwood was common also in the bouquets carried by churchgoing women in the province of Dalecarlia in the middle of Sweden. In addition, yet a peculiar custom existed there. In some parishes in the middle of the nineteenth century, it was common for women to chew onions *Allium cepa* L., during church service in order to stay awake. The custom was observed by several visitors and was sometimes described as barbaric, and the smell hard to bear. Still the women seem to have appreciated the taste and aroma [19, 59].

The occurrence of local variation may be observed. In some parts of central Sweden, for instance the province of Härjedalen, the church visitors brought the scented bracket fungus *Haploporus odorus* (Sommerf.) Bondartsev & Singer, to sniff during the service [52, 65]. In the subarctic northernmost Sweden, churchgoing women often carried scented grasses instead of herbs, especially *Anthoxanthum odoratum* L. and *Milium effusum* L. This is also known from Dalecarlia, as observed in Nås parish by Linnaeus in 1734 [52, 66, 67].

The custom of chewing and sharing spruce resin, *Picea abies* (L.) H. Karst, was another way for the peasants to entertain themselves during the service. This custom of chewing raisin in the church is especially identified with Dalecarlia. The resin was gathered in particular by the herding maids who spent the summer with the cattle in the mountains, and it was given as gifts to the boys when they arrived back at the villages in the autumn [68–71].

### Function of the church bouquet

After a long week of heavy work with few opportunities to rest, the churchgoers drifted asleep during the long, boring sermons. The aromatic herbs, onions, scented grasses and resin had one essential function—to keep the churchgoers awake. While the women were smelling the herbs, the men chewed tobacco, snuff or even shared strong liquor. A churchwarden made sure that they stayed awake by using a stick or even a whip. However, there were also other explanations for their use [72]. From the Reformation to the end of the nineteenth century, the men sat on the right-hand side of the church during services and the women sat on the other, i.e. the left-hand side. We may conclude that the use described was primarily a women’s custom [2, 7].

If the season allowed, the women carried a bouquet of fragrant plants of three species, referring to the Trinity, according to one record. The twig could also serve as a bookmark. It was put in the hymnbook to mark the day’s text and moved as the church year progressed. It can also be noted that southernwood and lavender leaves have been used since a long time ago to prevent the destruction of books by insect pests [2, 7, 73].

The custom of church bouquets thus testifies the peasant women’s knowledge of plants, of which we nowadays only have limited knowledge ourselves. The fragrant herbs typically carried by the women corresponded to the snuffbox carried by the men seated on the other side of the church. The bouquet, the resin, and the bottle of spices played an important role in expressing solidarity and friendship on the women’s side. It was a form of closeness that was lacking on the men’s side where one of them would at most offer his benchmate some snuff or a shot of aquavit. The women offered candies to their bench neighbours, shared their herbs and sent round the bouquets and fragrance bottles. The women’s side also stood for beauty and fragrance. One informant recalled that in the past, “it was lovely in the church, it smelled wonderfully of lavender and southernwood” [7, 74, 75].

For the youth, the church bouquet has also served as an amorous item of exchange, not least at the special youth worship services. On these occasions, the young people had large flower brooms with them, which were swapped when their homeward roads separated them from each other. In this way, the herbs became an important element of the communication between the sexes. Flower brooms could also be exchanged during the worship service. At a parish meeting in the eighteenth century province of Småland, complainants were displeased that the youngsters had thrown flower bouquets at each other during the service [64]. The custom of church bouquets made its entrance among the young people at their confirmation [7].

### Contemporary use

In the national inventories in Sweden, southernwood stood out as a relatively common heirloom plant that had been shared among garden owners outside the commercial distribution paths, spread from hand-to-hand [20]. In several cases, the southernwood had been grown within a family for generations and carefully cared for and transplanted when the household moved within the country. Southernwood was often described as being connected with positive memories of the scent and stories about the use of the herb in church by earlier generations [20].

**Fig. 4:**
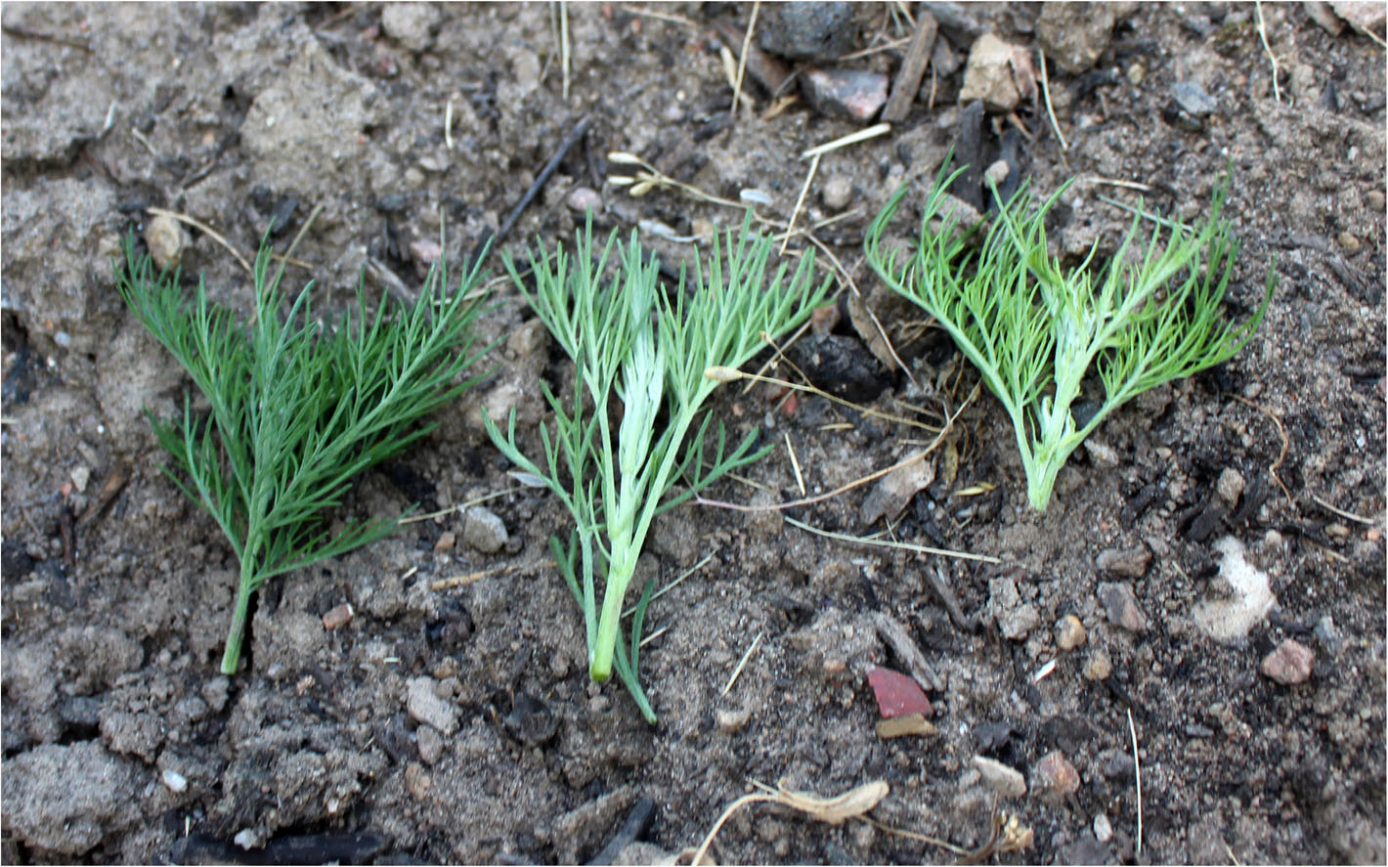
New growths in May 2019 with different hues of three genebank accessions of Swedish southernwood. From left to right “Söbben”, “Abbröt” and “Predikoväcka” (Photo Erik de Vahl)

**Fig. 5:**
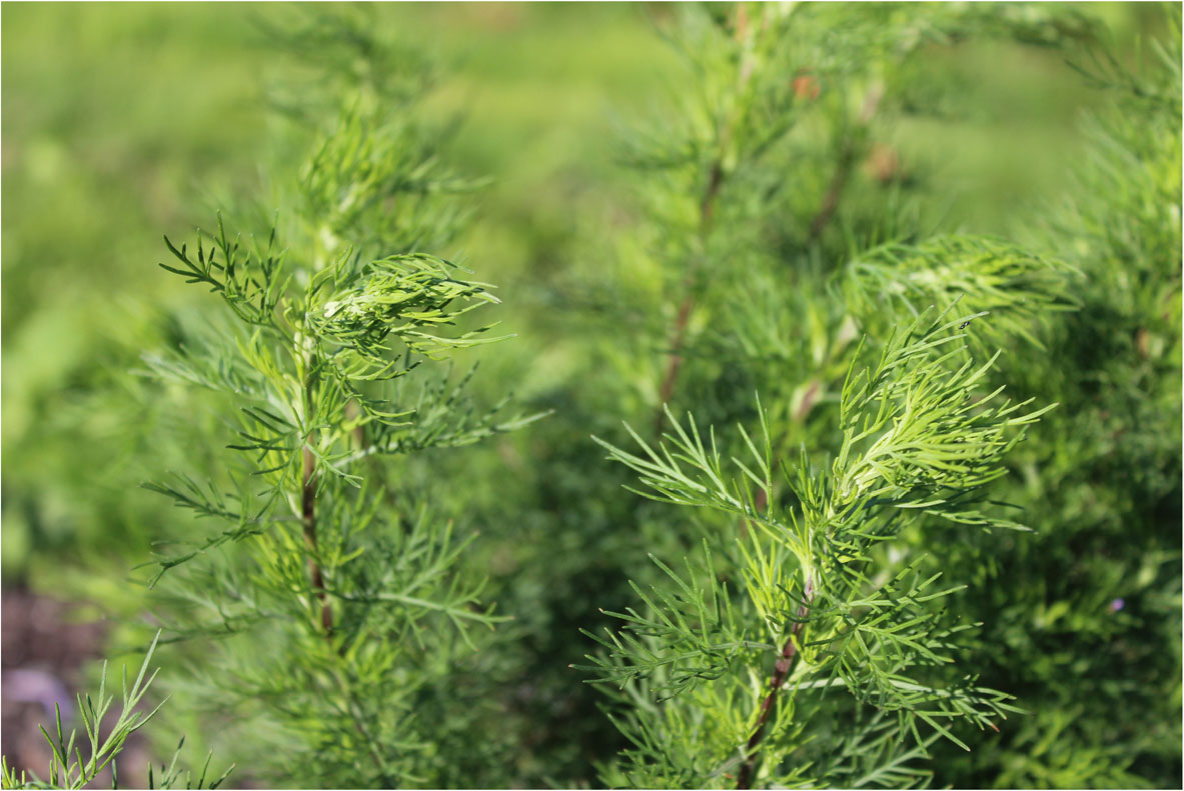
*Artermisia abrotanum* “Predikoväcka” from the province of Dalarna is horticulturally appreciated for its pleasant scent and growing habit. It was found in the national inventories for cultivated plants and has been introduced as a cultivar (Photo Erik de Vahl)

One of the accessions collected in the inventories has been released as an heirloom cultivar with the name *Predikoväcka* “wake up during sermon” through the trademark “*Grönt kulturarv*” (“Green Heritage”) established by the Programme for Diversity of Cultivated Plants to promote the use of cultural heritage plants with documented histories (Figs. 4 and 5).

Although sometimes recommended as a plant to flavour aquavit or as a spice in food, it is nowadays hardly ever used by Swedish consumers. Currently, one often encounters the species in contemporary Bible gardens, monastery museum gardens (e.g. Gudhem, Nydala, Vadstena, and Vreta) and other herbal gardens with historical plants, which have been established for educational and museological purposes [76].

### Cultivars

In Sweden, no known named cultivars were commercially available in the early 1900s, not even by growers who specialised in medical herbs. Because of the climate, no sexual propagation of the species is possible in the Nordic countries, but the material collected from the inventories organised by the Programme for Diversity of Cultivated Plants still showed a remarkable morphological variation (Fig. 3). Difference in earliness, colour, scent and growing habit were evaluated and described [20].

One of the accessions, *abbröt*, named after the dialectal pronunciation of the common name in Western Sweden, morphologically resembles a variety which is occasionally spread in the trade as *Artemisia alba* “Cola”. This form with its light green and robust foliage and pleasant low camphor scent was found in several gardens in the Swedish inventories and was always known and grown as Southernwood, *Artemisia abrotanum*. The taxonomic status of this form is still to be evaluated.

Even though no cultivars are known from old garden literature, a few records of popular, rural knowledge can be found regarding different qualities of the species’ plant material. From the province of Blekinge, one of the several respondents born in the 1850s and 1860s describes how southernwood was used by her mother as a dried herb mixed in the blood when making blood sausage. The respondent refers to the southernwood used as “the green kind” and points out that the light one was not good enough. She also refers to one of the forms as “German Southernwood” [77].

## Discussion

The custom of church bouquets reflects the peasantry’s multifaceted relationship with plants. It reveals the women’s knowledge of specific plant species, especially those kept in the allotments because of their scent. It also illustrates the earlier beauty ideal. The whole complex of practices demonstrates that plants therefore had a broader function than merely preventing sleep during services. The church bouquet was part of the female appearance in the pre-industrial peasant society and gave stimulation and colour to the relationship between the sexes, as written by sociologist Berndt Gustafsson [7].

One may ask why the peasant women were particularly interested in southernwood and other fragrant herbs. Local historian Linnar Linnarsson explains that the peasant women’s interest in southernwood was for its refreshing scent and its long-lasting availability for use in allotments close to the residential house all summer. In addition, memories of the practice and care of these herbs by their mothers and grandmothers added value for these women. Southernwood was connected with the church service and helped them in their worship and reminiscences. In addition, the bouquet was something that was relaxing and kept their hands occupied [78].

However, few people regularly attend church services nowadays, and hardly anyone brings a church bouquet. Therefore, it is very interesting that *A. abrotanum* is still grown in older gardens as a memory of its former use. It is also obvious that memories of former uses documented in the inventories conform to ethnobotanical records. Southernwood were found to be the most common plant in the church bouquet (see above).

The Convention on Biological Diversity, CBD, which entered into force in 1993, emphasises the importance of the conservation of biological diversity by each country [79]. Sweden’s National Programme for Conservation of Cultivated Diversity (POM) was a response to the convention and defined a need not only to preserve the biologically important plant material that is essential for meeting food security, plant breeding and research, but also to include horticulturally and culturally important plant groups within the national programme [80]. The National Gene Bank for vegetative propagated horticultural plants has the task of preserving not only the plant material but also the documentation that links the immaterial cultural heritage of the specific accessions with the conserved genotypes/accessions [81].

Preserving cultivated cultural and domesticated plants that are dependent on continuous cultivation requires ex situ conservation. Gene bank accessions of culturally and historically valuable plants without clear links to biological values, such as southernwood, require in-depth knowledge of how values connected to cultural heritage should be formulated and understood. These values are sometimes also categorised as cultural ecosystem services and described as including aesthetic enjoyment, recreation, artistic and spiritual fulfilment, and intellectual development, but sometimes also national economic values [82, 83]. Another approach in the understanding of non-biological values of cultivated plants might be found in the term bio-cultural diversity as formulated by Maffi [84]. The label combines biological, cultural, and linguistic diversity in a way that enables an understanding of humans and human cultures not only as a threat to the biological diversity but also as creators, enchanters and maintainers of it.

The Swedish national programme not only has the assignment to conserve the green cultural heritage, but also to utilise it. This includes the reintroduction of plants and seeds into the market. The cultural ecosystem services category includes both economic and cultural aspects that correspond to this dualist task of the programme [85]. Southernwood is a suitable example to illustrate how aspects, historically understood as social values linked to cultural customs, recreation and contemplation, today must be regarded as cultural-historical values that could also be understood as part of a past bio-cultural diversity [86]. The task of utilisation of the preserved plant material by means of its reintroduction into the market might be a way of creating new, contemporary social and economic values. These values are then highly dependent on the knowledge and documentation of cultural-historical values linked to the crop, species or cultivars, as well as to garden-historical knowledge of regional customs and old growing systems.

## Conclusion

The documentation of the Swedish inventories highlighted how childhood memories of customs linked to older relatives were often strongly associated with specific plants preserved by the donors. The tradition of sharing cuttings of plant material not usually found in garden centres or stores, from person-to-person, has intertwined old cultural historical values with contemporary social values linked to social networks and the plant trade.

Through the creation of the Grönt kulturarv® trademark, translated as “Green Heritage”, the Swedish National Programme, in collaboration with commercial operators, has supported the launch of several old cultivars from inventories. The utilisation of these garden plants, including southernwood, contributes to strengthening the economic and social values of the conserved plant material. At the same time, it complicates the conservation of the cultural-historical aspects associated with the non-commercial, person-to-person sharing in small communities. If future restoration work of old gardens uses the same limited range of reintroduced and historically correct cultivars available in the trade, it might affect the future diversity of cultivated plants.

The fact that only a fraction of the plant material conserved ex situ in field gene banks is commercially reintroduced means that the large diversity identified and described in the inventories instead might be remedied in future gardens. To further discuss the values of preserved plant material, sometimes understood and described as cultural ecosystem services or as part of the bio-cultural diversity, we need to deepen the link to scientists working in different fields connected to the plant material.

The results show that southernwood has been important both as probably the most common herb used in the bouquet by female rural churchgoers and later as a memory of the customs of former times. It was once an important part of women’s culture, and today it is an imagined fragrant memory of an earlier time.

## Data Availability

All data generated or analysed during this study are included in this published article.
